# Customizing egg incubators for Cameroon: A design and construction guide

**DOI:** 10.1371/journal.pone.0322357

**Published:** 2025-05-15

**Authors:** Marck Jickel Kemegne Tagne, Thomas Kanaa, Paul Etouke Owoundi, Arnaud Obono Biyobo

**Affiliations:** 1 Computer and Automatic Engineering, H.T.T.T.C Douala (University of Douala), Douala, Cameroon; 2 Department of Electrical and Telecommunications Engineering, University of Yaoundé I, Yaoundé, Cameroon; Ain Shams University Faculty of Agriculture, EGYPT

## Abstract

In Cameroon, poultry farming represents an important source of income for many families and is a key sector for economic development and food security. However, there is a deficiency in suitable infrastructure, especially high-performance and affordable incubators, leading many poultry farmers to resort to manual incubation techniques, which are often inefficient and labor-intensive. This paper aims to build an automatic incubator using locally accessible materials, optimized techniques, and modern, simple technologies. This paper also serves as a construction guide. Its initiative offers a significant opportunity to improve poultry farming practices, increase local productivity, and contribute to sustainable development. The incubator is built using the prototyping method, and the optimization of energy efficiency for the system was achieved through the mathematical modeling of heat transfer. The study’s findings indicate that the incubator is a dependable and effective solution for hatching poultry eggs. Its user-friendly design, ease of maintenance, and affordability make it an excellent choice for local poultry farmers.

## 1. Introduction

Egg incubation plays a crucial role in poultry farming, directly influencing the productivity and quality of chicks. In Cameroon, poultry farming represents an important source of income for many families and is a key sector for economic development and food security. However, suitable infrastructure is deficient, especially high-performance and affordable incubators, leading many poultry farmers to resort to manual incubation techniques, which are often inefficient and labor-intensive. The design of an automatic incubator, which uses locally accessible materials, optimized techniques, and modern and simple technologies, offers a significant opportunity to improve poultry farming practices, increase local productivity, and contribute to sustainable development.

An incubator is a specific apparatus that supports and maintains microbiological and cell cultures. It carefully regulates factors such as temperature, humidity, and atmospheric gases, including CO_2_ and oxygen, to establish the perfect environment. These incubators are crucial instruments in areas such as cell biology, microbiology, and molecular biology, enabling the cultivation of both bacterial and eukaryotic cells for different experimental objectives [1].

**Fig 1:**
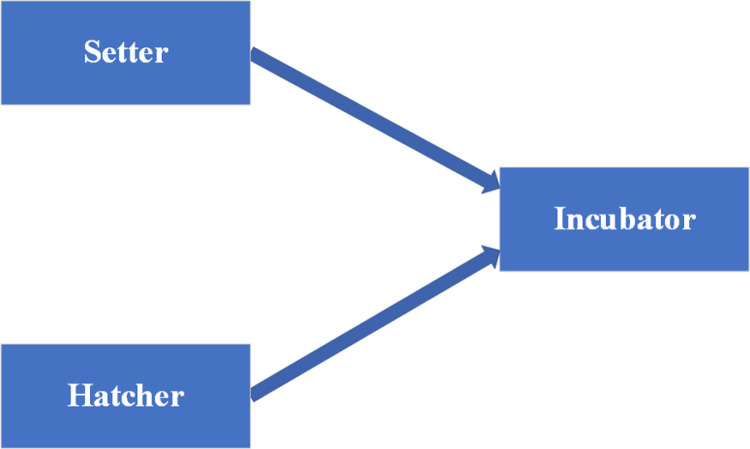
Parts of an egg incubator.

In the poultry industry, incubators frequently replace hens [2,3], leading to increased hatch rates due to precise control over temperature and humidity. While commercial breeders have access to a variety of incubator brands, small-scale and backyard poultry farmers often rely on natural incubation or traditional incubators. These methods can be limited in effectiveness, inadequate, or stressful. This problem has attracted the attention of numerous researchers, including [2–4], but the current state of research in Cameroon shows that it remains a challenging issue. Some of the key challenges include the high cost of commercial incubators, a shortage of modern technology, high energy consumption, and a lack of detailed guidelines. This article will, therefore, focus on using locally available materials, optimizing energy efficiency, and providing practical guidance for building incubators, making this solution more accessible to a wider audience. Generally, an egg incubator is made up of two parts; a setter and a hatcher, as shown in Fig 1.

## 2. Conditions for achieving successful incubation

The capacity of an egg to hatch is primarily determined by the environment during incubation. Temperature, humidity, ventilation, and egg rotation during the incubation phase all have a major impact on the quality and hatchability of fertile eggs [5–8]. These characteristics were considered throughout the design of the incubator.

Among the aforementioned variables, temperature is the most significant and can significantly affect the hatchability of viable eggs [9,10].

In the incubator, the temperature range, humidity level, and air mass flow rate are connected variables. Controlling the egg incubator’s interior temperature also regulates its relative humidity [11,12].

The relationship between the temperature of the developing embryo, the temperature of the incubator, the heat production of the embryo, and the thermal conductivity of the eggs and their surroundings were described in [5,13] via a straightforward mathematical model, as shown in Equation 1 [14].


$$T_{egg}=T_{inc}+\frac{\left(H_{emb}-H_{waterloss}\right)}{K}$$ (1)

**Equation 1: Temperature of the egg (Celsius)**

Where,


$$T_{egg}=Temperatureoftheegg(Celsius)$$

$$T_{inc}=Temperatureofincubator(Celsius)$$

$$H_{emb}=Heatproductionofembryoatagivenmomentofincubation(Watts)$$

$$H_{waterloss}=Heatlossfromevaporativecooling(Watts)$$

K=thermalconductanceofeggandsurroundingboundaryofairaroundtheegg

(WattsperdegreeCelsius)

According to [12] The air in the incubator needs to be changed approximately eight times a day or once every 3 hours for adequate ventilation. This same quantity of air within every 3 hours contains the heat energy required to increase the temperature of the egg to the desired temperature.


$$V_{fan}\times A_{T}=\frac{V_{chamber}}{T_{safe}}$$ (2)

Where,

$V_{fan}=Speedofventilationfaninmm/s$

$A_{T}=Totalcross-sectionalareaoftheventilationholes$

$V_{chamber}=Volumeoftheincubatorchamber$

$T_{safe}=Safetimerequiretoemptyalltheairinthechamber$

This can also be considered the size of the minimum ventilation hole needed to ensure proper ventilation of the eggs in the incubator.

### 2.1. Temperature exchange

**Fig 2:**
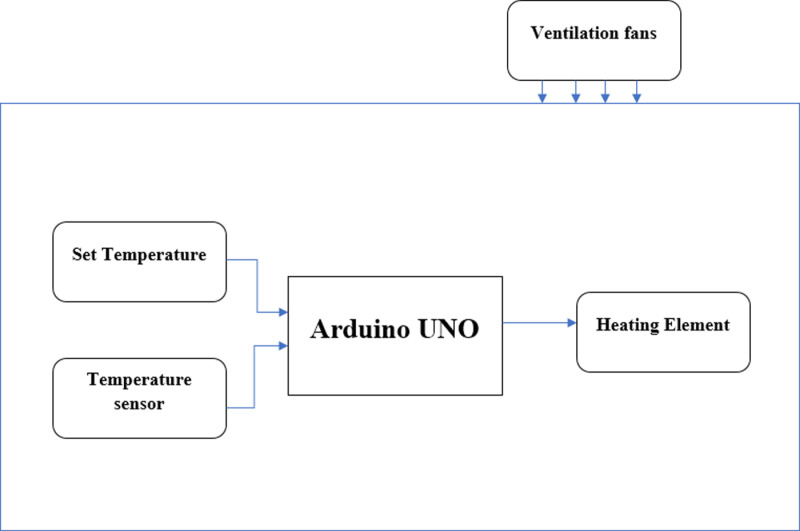
Temperature control block.

Temperature exchange is of crucial importance for incubation, Fig 2 highlights how temperature is controlled by the Arduino Uno. The *set temperature* is the temperature entered by the user, and the *temperature sensor* is the actual temperature of the incubating space (this temperature value is obtained from the sensor).

- The heat required to increase the temperature of the egg from 25^◦^C to 38^◦^C

$$Q_{egg}=m_{egg}c_{egg}\Delta T$$ (3)

Where,


$$Q_{egg}=quantityofheatrequiredtoraisethetemperatureofeggfrom25^{\circ }\text{C}to38^{\circ }$$

$$m_{egg}=massoffeggs(Kg)$$

$$c_{egg}=specificheatcapacityofegg(KJ/KgK^{-1})$$

ΔT=temperaturedifference(K)

Using, $c_{egg}$= $3.313KJ/KgK^{-1}$ [15].

An egg’s weight varies between 45g and 75g depending mainly on the age of the hen and, to a lesser extent, on its genotype [16]. Thus, we will use an average weight of 60 g = 0.06 kg.

- Heat production by the eggs:

Eggs, in their period of development, perform metabolic activities and produce heat according to [17]. The egg heat production rate ranges from 137 mW to 155 mW for small and big eggs, respectively. A heat production rate of 146 mW, which is the average, is used in this work.

- Heat required to increase the temperature of the air from 25^◦^C to 38^◦^C

$$Q_{air}=m_{air}c_{air}\Delta T$$ (4)

Where,

$Q_{air}=quantityofheatrequiredtoincreasethetemperatureofairfrom25^{\circ }\text{C}to38^{\circ }\text{C}$

$m_{air}=massoffair(Kg)$

$c_{air}=specificheatcapacityofair(KJ/KgK^{-1})$

ΔT=temperaturedifference(K

Using, $c_{air}$= $1.004KJ/KgK^{-1}$[15].

- The quantity of heat lost by the ventilation hole is as follows:

The quantity of heat loss can be calculated via the following formula


$$Q_{v}=\rho V\Delta T$$ (5)

Where,

$Q_{v}$ = Quantity of heat loss by the ventilation hole

*V = Ventilation rate(* $m^{3}s$ *) = (0.0192554582) = 0.0385, since we have two fans*

ρ = density of air = $1.11202kgm^{-3}$

**Fig 3:**
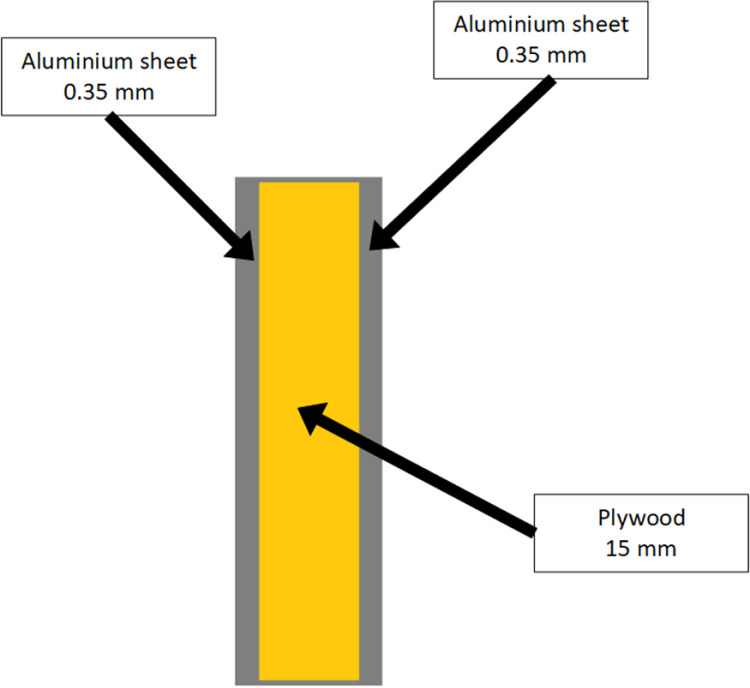
Wall of our incubator.

- The quantity of heat loss through the walls during incubation is as follows:

To minimize heat loss, the wall of our incubator is composed of three layers: the inner and outer layers are both made of aluminum sheets, while the middle layer is plywood. This configuration is illustrated in Fig 3.

Since our incubator is made up of an adiabatic wall, the heat loss is negligible; thus, $Q_{wall}=0W$

- Total heat loss from the incubator system

The total heat loss can be obtained using the following equation (Equation 6).


$$Q_{LT}=Q_{egg}+Q_{air}+Q_{v}+Q_{wall}$$ (6)

Where,

$Q_{LT}$ = total heat loss in the incubator chamber, W

$Q_{egg}$ = heat energy required to increase the temperature of the egg from 25 °C to 38 °C, W

$Q_{air}$ = heat energy required to increase the temperature of the air from 25 °C to 38 °C, W

$Q_{v}$ = heat loss by ventilation hole,W

$Q_{wall}$ = heat loss through the wall, W

## 3. Materials and methods

**Fig 4:**
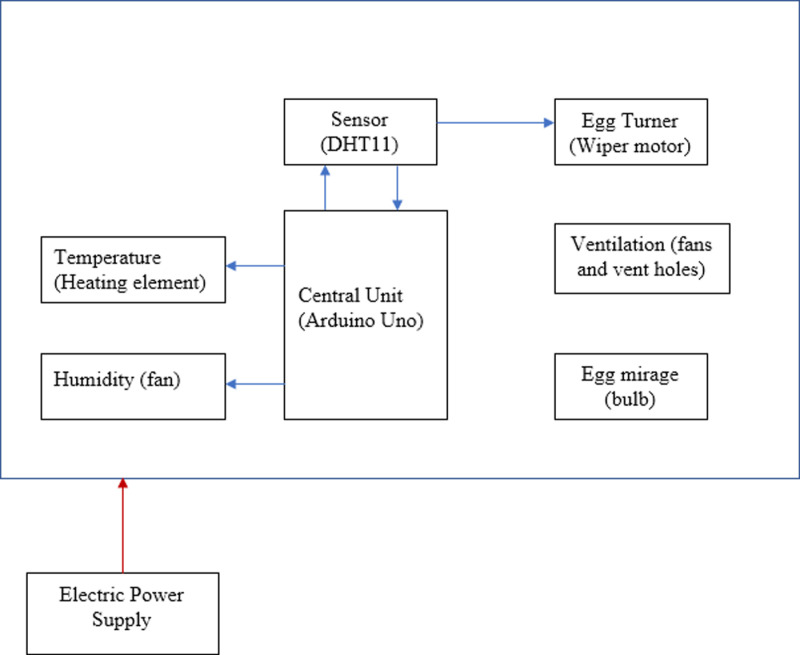
Block diagram of the incubator.

The incubator system features automated subsystems for temperature, humidity, ventilation, and an assisted egg-turning mechanism, which create the optimal conditions for efficient performance [6]. All subsystems were analyzed to guarantee the precise identification of their appropriate values. The incubator, categorized as a thermal system, entails both the circulation and retention of heat. Therefore, it is essential to sustain a certain level of heat throughout its operational duration. Likewise, the humidity levels fluctuate. The heat transfer in this process typically happens through the methods of convection, radiation, and conduction. The convection and radiation processes, assisted by fans, promote uniform heat distribution within the incubation chamber. Additionally, a key function of the system is the turning mechanism that rotates the eggs when activated by the user. The block diagram of the system is illustrated in Fig 4.

### 3.1. Materials

The table below (Table 1) List all the materials (their price, description, and quantity) used to build our egg incubator. Each item was chosen based on its local availability (how easily an individual can get it), its price (its affordability), and its ability to fulfill technical requirements (efficiency).

**Table 1:** List of materials.

N°	Material	Description	Quantity	Total price/ USD
1	Display	LCD 2004 with I2C module	1	8.84
2	Heating element	100 W	1	5.63
3	Relay module 4 chains	5DCV, 10A 250VAC, 15A 125VAC	1	9.65
4	Breadboard	European standard 830 holes test plate	1	3.22
5	Arduino microcontroller	Arduino Uno R3	1	11.25
6	Temperature and humidity sensor in module	DHT11	1	3.22
7	Arduino power	cable with 9 V battery output	1	0.48
8	Connectors	60 cables Male/ Male (20 centimeters)	1	2.41
9	Pre-perforated plate	9 * 5 cm	1	0.96
10	Fan	12 DC Brushless	4	12.86
11	Power adapter	12 VDC	1	8.04
12	Soldering Iron	220V - 60W	1	3.22
13	Glue gun	110 V/220 V – 60 W	1	7.23
14	Refill for glue gun	20 Cm length	6	1.93
15	Mini switch	250 V 3A	2	1.21
16	Soldering Paste	Advance quality ZJ-18	1	1.13
17	Soldering tin	Tin 63/37	1	0.80
18	Male to Male 40 Pin	Connector Row- 2.54mm	1	1.13
19	Big buzzer	12 V	1	0.80
20	LED	5mm – 3mm	1	0.080
21	Male to Female 40 Pin	Connector Row- 2.54mm	1	1.45
22	Limit switch	4A 125 AC	2	0.64
23	Resistor	10K	5	0.40
24	Potentiometer	B50K	1	0.40
25	Transistor	P75NF758 C706Q B	1	0.80
26	Big push buttons with cap	Big push button with hat	6	0.96
27	Cabinet	Plywood and aluminum sheet	1	56.26
28	Support for Aluminum Egg tray support	Iron	1	17.68
29	Aluminum Egg tray support	Aluminum	4	8.04
30	Wiper motor	12VDC Valeo wiper motor	1	6.43
31	Silicon glue	Insulating glue	1	2.41
**Total**				**177.63 USD**

### 3.2. Methodology

The prototyping method, the method used by the authors, is a systems development method in which a prototype is built, tested, and then reworked as necessary until an acceptable outcome is achieved from which the complete system or product can be developed. This model allows rapid creation of prototypes; By developing prototypes, the development team gains a better understanding of the client’s requirements; Users can provide early feedback, which speeds up the development process; Even with limited initial requirements, the team can start the development process and refine the solution based on ongoing feedback; An iterative feedback loop ensures that the final product aligns with user needs; The prototypes developed during the process can be reused for similar projects, which saves time and effort in subsequent development cycles; Developing prototypes helps identify errors and missing features early in the project, and corrections can be made before the final product is built, reducing risks.

The different items used for the construction of this incubator were based on their local availability, affordable price, simplicity of implementation, and capacity to meet the technical requirements.

#### 3.2.1. Incubator structure.

The cabinet has a height of 1230 mm, a width of 510 mm, and a length of 540 mm. It is made up of an adiabatic wall composed of 15 mm plywood covered on both sides with an aluminum sheet 0.35 mm in thickness.

The aluminum egg tray support was built as indicated with aluminum (angle of size 20 mm) because it’s light, which is solid enough to carry the plastic egg tray, and it’s a fast energy conductor and giver. It is 330 mm X 300 mm in size. In such a way, the egg tray fits exactly in the direction of turning of the motor so that it does not slide and fall.

**Fig 5:**
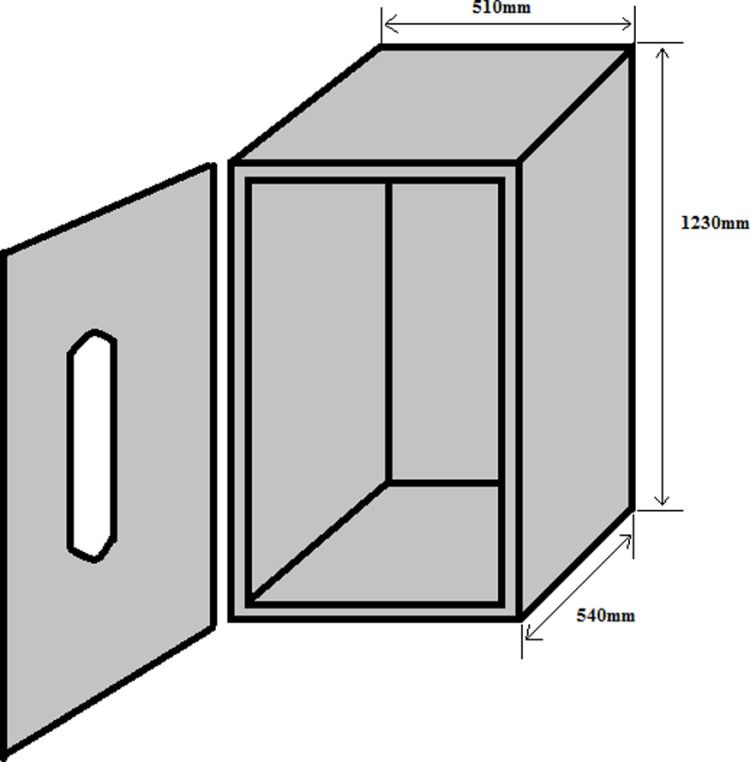
Incubator cabinet.

**Fig 6:**
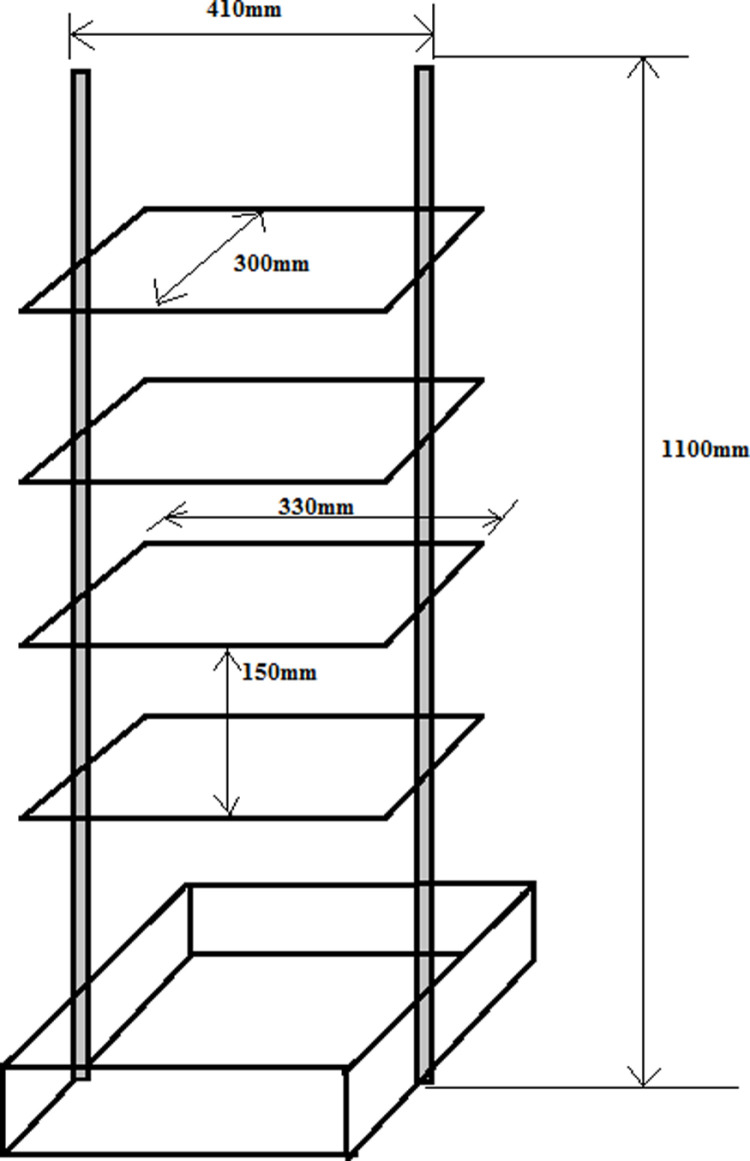
Incubator Support for aluminum egg tray support and motors.

The support for the aluminum egg tray support and motor had to be solid enough to support a weight of approximately 15 kg. For this reason, it was built with an iron angle of 25 mm. The other dimensions are expressed in Figs 5 and 6 below.

#### 3.2.2. Ventilation system.

**Fig 7:**
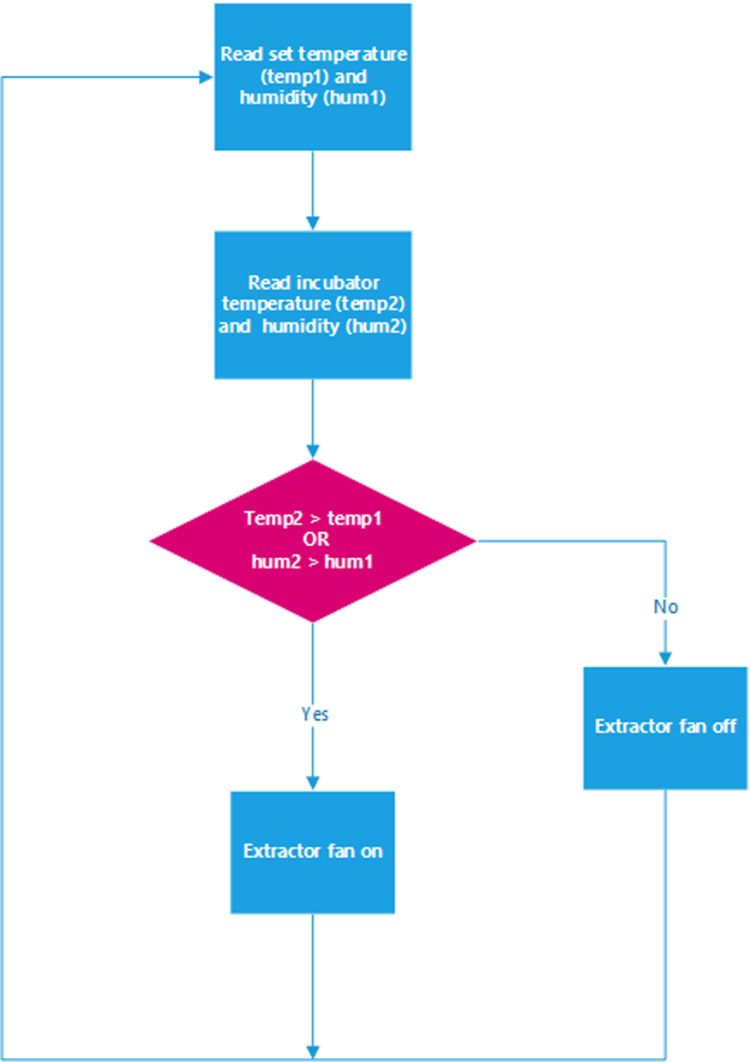
Flowchart of exhaust fan.

Eggs are living organisms that require oxygen to perform their metabolic activities. It is thus important to maintain proper oxygenation as it may impact the hatching percentage negatively if the eggs are not well oxygenated. The ventilation system is made up of ventilation holes, an exhaust fan, and ventilation fans to ensure the proper distribution of oxygen, heat, and humidity. The exhaust fan, in the case of excess humidity or overheating, pulls in fresh air to set the humidity or temperature to set points. The flowchart illustrated in Fig 7 describes how the ventilation system operates. Temp1 and hum1, and temp2 and hum2 are respectively set and sensed values.

#### 3.2.3. Temperature control system.

**Fig 8:**
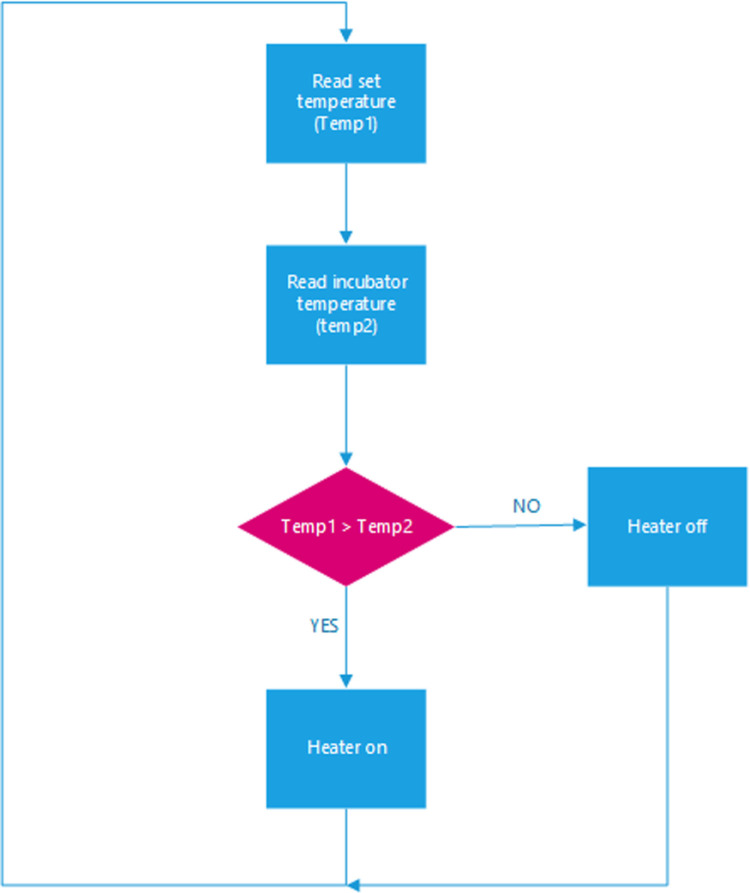
Flowchart of the heating system.

**Fig 9:**
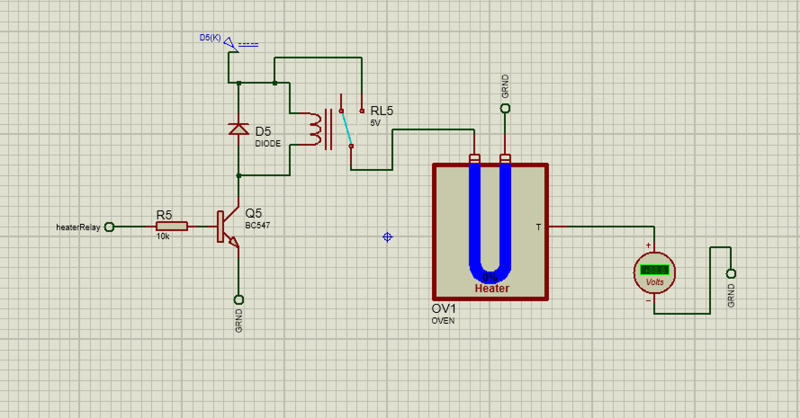
Schematic diagram of the heating subsystem.

In our incubator, heat is maintained at a set point via the control system by the user. The heat is produced by a heating element of 100 W 12VDC. When the user sets a temperature value (Temp1 in Fig 8 for the incubator via the user interface, the value is stored. If the set temperature is greater than or equal to the sensed temperature (the actual temperature of the incubator interior, sensed by the DHT11 sensor, Temp2), the heater is turned off otherwise, the heater is turned on. The temperature relay is used to toggle the temperature on and off. The controls are illustrated by the flow chart in Fig 8. Fig 9 illustrates the schematic diagram of the heating subsystem.

The choice of the DHT11 was conditioned by its price, ability to perform the required task, and most importantly, its readily local availability.

#### 3.2.4. Humidity control.

**Fig 10:**
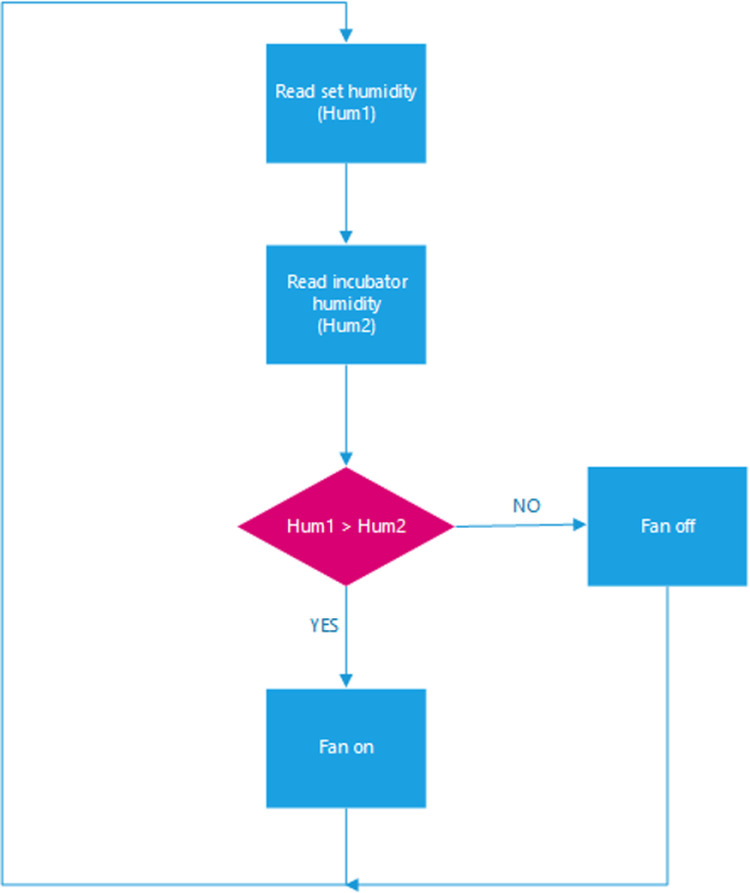
Flowchart of the humidity system.

**Fig 11:**
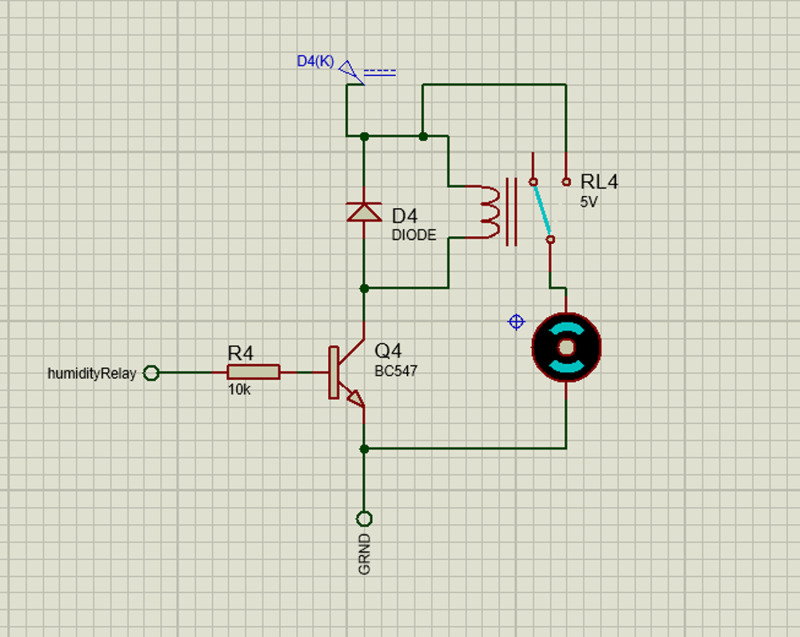
Humidity subsystem schematic diagram.

In our incubator, humidity is maintained at a set point via the control system by the user. The humidity is controlled by a 12 VDC brushless fan (evaporative humidification). The fan is placed on a water tank. When the user sets a humidity value (Hum1), Arduino Uno compares the set humidity with the humidity value (Hum2) sensed by the DHT11 sensor. If the humidity set by the user is greater than or equal to the sensed humidity, the fan is turned off; otherwise, it’s turned on. The humidity relay is used to toggle the temperature on and off. The control is maintained as indicated by the flow chart in Fig 10. Fig 11 illustrates the schematic diagram of the humidity control subsystem.

#### 3.2.5. Egg turner system.

**Fig 12:**
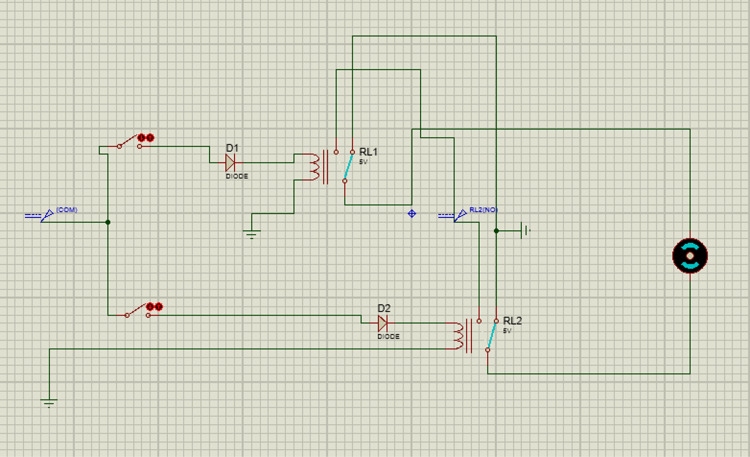
Schematic of the basic functioning of the turner system.

The motor can rotate in both directions by reversing the direction of flow of current through it thus causing forward and backwards movement of the egg tray. Its schematic diagram is shown in Fig 12.

#### 3.2.6. Coding (Programming the Arduino Uno).

The Arduino microcontroller necessitates an understanding of the Arduino programming language (which is based on wiring) and familiarity with the Arduino IDE. The Arduino programming language resembles the C language and features a compiler that is beginner-friendly while also offering enough flexibility for more experienced users. The programming involved setting up the LCD, the ventilation control system, the temperature control system, the humidity control system, the control buttons, the alarm system, and the egg-turning system according to the control logic illustrated by the flow charts in this document.

The program can be logically divided into 3 main parts: Variable declaration and Initialization, Function declaration and definition, and the program’s main entry.

**3.2.6.1. Variable declaration and initialization:** In this section, all the necessary variables required for the smooth execution of our program are created, and appropriate initial values are assigned to some of them. These values could include Arduino PINs or simple constants. Specifically, the variables for managing:

- Temperature (temperature_user,...),
- Humidity (humidity_user, …),
- Relays (alarmRelay, heaterRelay, humidityRelay, motorRelayF - Front, motorRelayB - Back, exhaustRelay),
- LCD (numOfScreens, currentScreen)
- Buttons (inputPins, inputState, …) are defined here with their appropriate values.

These variables are essential for the program to interact with the various hardware components like sensors, relays, LCD, and buttons.

**3.2.6.2. Function declaration and definition**: In this section, the different functionalities of the incubator were divided into modules. Each has a name and an implementation. The aim is to ease development and maintainability. Among these functionalities. The key functions include:

- Setup: This function initializes the relays and the LCD screen.
- displayReadings: This function provides the default LCD screen display.
- parameterChange: This function permits changing the values of parameters on the current LCD screen.
- checkAndSwitchRelays: This function compares sensor values with set values to switch relays automatically.
- setInputFlags: This function provides the code to initialize the control buttons.

These functions encapsulate specific tasks, allowing for organized and modular code. They interact with the declared variables and control the hardware components based on the logic implemented within them.

**3.2.6.3. Program’s main entry**: The loop() function runs continuously after setup() has finished. It’s where the main logic of the program resides, enabling the program to perform repeated tasks. Within the loop() function, other functions are nested and executed when specific conditions are met or events are triggered. Here’s how the interactions happen:

- **Reading Sensor Values**: The program continuously reads sensor values for temperature and humidity.
- **Managing Temperature and Humidity**: Based on the sensor readings, the program executes a section based on specific condition validation in the *checkAndSwitchRelays* function to control the relays (e.g., turning on/off the heater or fan, turning on/off the buzzer).
- **Updating LCD Display**: The program updates the LCD with the current readings and any changes in parameters, *displayReadings* function.
- **Handling User Inputs**: The program checks for user inputs via buttons and allows changes to the parameters using the *parameterChange* function.
• **Automatic Relay Switching**: The checkAndSwitchRelays function is called to switch relays automatically based on the sensor readings and set values.

This continuous loop ensures that the incubator maintains the desired conditions by constantly monitoring and adjusting the parameters.

*See the appendix for the code in* S1 File.

## 4. Results and discussion

### 4.1. Results

**Fig 13:**
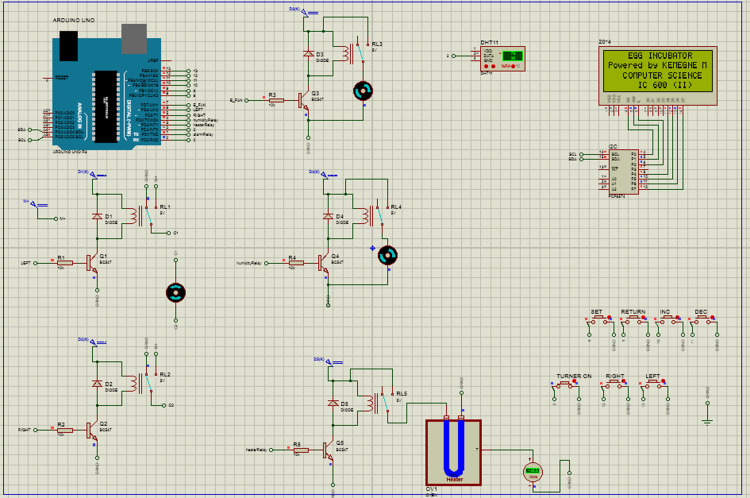
Proteus stimulation of the incubator.

After confirming the proper functioning of the egg incubator simulation (performed in Proteus 8 Professional IDE), physical development was carried out. The simulations were conducted to ensure all components operated as expected. These simulations included: measurements of heat and humidity, visualizing the menu and managing parameters on LCD, responding to triggered buttons, monitoring motor direction upon trigger, and the behavior of the alarm and exhaust fan. The simulated results allowed us to refine the code to demonstrate that all components functioned as expected. The schematic diagram of the egg incubator is shown in Fig 13.

**Fig 14:**
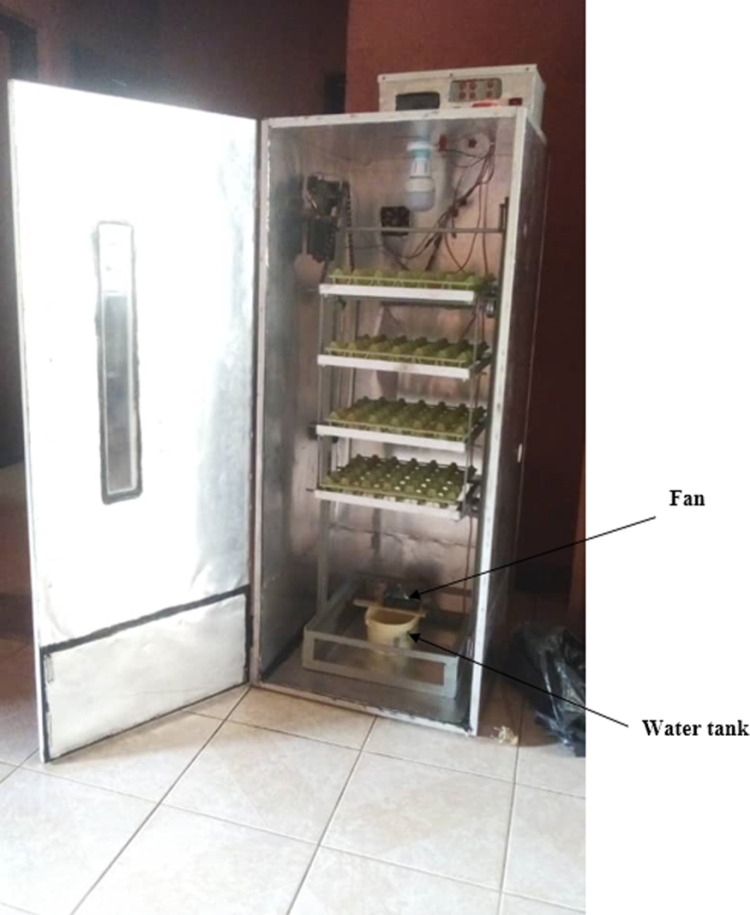
The egg incubator.

With the encouraging results from the simulations, we proceeded to construct the physical incubator using the materials outlined in the design. The construction process involved assembling a three-layer wall (aluminum sheets for the inner and outer layers, with plywood as the middle layer), integrating the temperature, ventilation, and humidity control systems, and installing the LCD for real-time monitoring. The initial setup included calibrating the sensors with user set values and verifying the responsiveness of the control system. The physical implementation is shown in Fig 14.

The system was observed for ten hours throughout the testing period, with the LCD screen displaying the temperature and humidity levels in the incubator at hourly intervals. This procedure enabled us to calculate the rate of heat rise from the initial incubator temperature to the steady-state temperature of 38°C.

Additionally, the monitoring allowed us to determine the incubator’s power usage by calculating the duty cycle of the heater and humidification fan.

**Fig 15:**
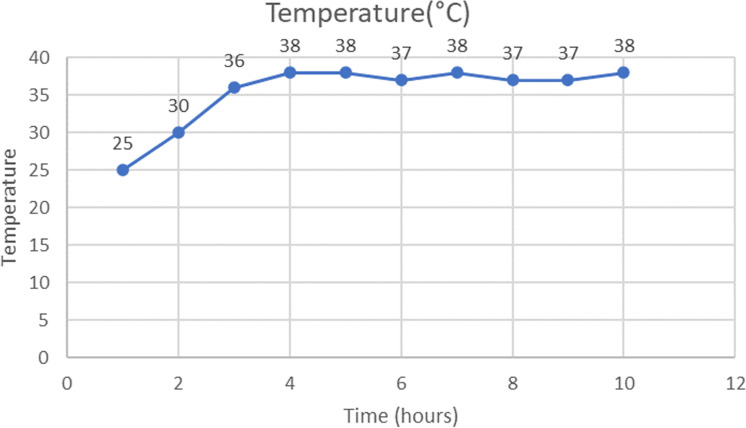
Evolution of temperature with time in the incubator.

**Fig 16:**
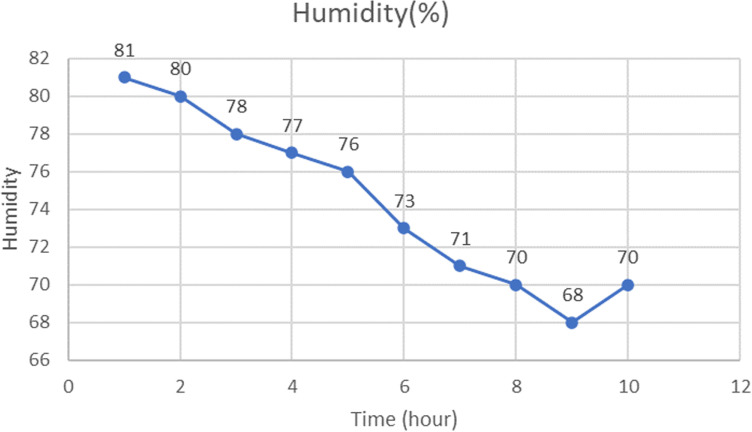
Evolution of humidity with time in the incubator.

- Temperature Change

- Fig 15 shows the rate of temperature change over time.
- The temperature in the incubator increased from 25°C to 37°C within the first 2 hours.
- After the initial 2 hours, the temperature stabilized near the set point value of 38°C, with minor fluctuations between 37°C and 38°C.
- The duty cycle of the heater is 70% (it should be noted that the incubator is empty).
- Despite variations in ambient temperature over the 10 hours, the incubator’s internal temperature remained relatively constant, indicating minimal heat loss through conduction.

- Humidity Change

- Fig 16 illustrates the rate of change in humidity over time.
- The internal humidity fluctuated between 75% and 68% from the recommended 70%.
- The duty cycle of the evaporative fan is 10% (the incubator is empty).
- This fluctuation further demonstrates the incubator’s ability to maintain the average relative humidity necessary for a successful hatch.

These results highlight the incubator’s efficiency in maintaining stable temperature and humidity levels, which are crucial for the successful hatching of eggs.

### 4.2. Discussion

#### 4.2.1. Comparative analysis of our incubator prototype and commercially available incubators.

In assessing our prototype incubator in comparison to commercially available options, various important aspects become apparent, such as price, efficiency, user-friendliness, availability of materials, and potential for customization. The following is an in-depth comparison:

**4.2.1.1. Cost**: *Prototype* ***i****ncubator:* The prototype was created with materials that are both locally available and budget-friendly, greatly lowering the total cost. It incorporated items like aluminum panels, plywood, and essential electronic components (such as sensors, relays, and an LCD screen), maintaining cost-effectiveness while still ensuring functionality.

Commercial incubators: Incubators available on the market typically have a higher cost because they utilize specialized materials and advanced technology. The inclusion of features like improved automation and precise control systems also adds to their elevated price.

**4.2.1.2. Performance**: Prototype incubator: Through both simulated and physical testing, the prototype demonstrated consistent performance in maintaining stable temperature and humidity levels, which are critical factors for successful egg incubation. It effectively manages air circulation, heat distribution, and humidity control, ensuring optimal conditions for hatching.

Commercial incubators: Commercial models often boast advanced temperature and humidity regulation systems, automated egg-turning mechanisms, and superior insulation. These features can lead to higher hatching success rates. Additionally, they typically include built-in safety mechanisms and more precise control systems, enhancing overall reliability. As example we have the HHD 200 Egg Incubator with an average price of 412.19 USD in Cameroon.

**4.2.1.3. Ease of use:** Prototype incubator: The prototype showcases a user-friendly design focused on simplicity, which allows local poultry farmers to use it easily. It comes equipped with an easy-to-read LCD screen and simple controls that enable users to monitor and modify only important parameters, ensuring smooth operation for those with minimal technical skills.

Commercial incubators: Although commercial incubators frequently feature advanced interfaces and enhanced functions, this complexity can make them difficult to operate. While they provide improved accuracy and automation, the intricate nature of these systems may lead to difficulties for users who are not well-versed in operating them.

**4.2.1.4. Availability of materials**: Prototype **i**ncubator: Utilizing materials that are both locally accessible and cost-effective guarantees that replacement components can be easily obtained and repairs can be performed with little difficulty. This ease of access is especially beneficial in areas with limited resources.

Commercial incubators: Commercial models often depend on unique or specialized parts, which can complicate and increase the costs of repairs. Replacement components may not be easily accessible in the local area and typically have to be sourced directly from the manufacturer, resulting in possible delays and higher expenses.

**4.2.1.5. Customization and flexibility:**

Prototype **i**ncubator: The design allows for customization based on local needs and preferences. Modifications can be applied to suit various kinds of eggs.

Commercial incubators: These typically provide reduced options for personalization. They are created to fulfill the needs of a wide range of users, which might not consistently correspond with particular local demands.

#### 4.2.2. Challenges in building such a project.

**4.2.2.1.** Understanding of electronics: Building an incubator often requires knowledge of electronics, such as how to wire and connect components such as sensors, heaters, and fans.

**4.2.2.2.** Programming skills: You must write code to control the temperature, humidity, and turning mechanisms.

## 5. Conclusion

Poultry farming in Cameroon is a vital source of income for many families and significantly contributes to the country’s economic development and food security. However, the sector faces a major challenge due to the lack of adequate infrastructure, particularly affordable and high-performance egg incubators. This deficiency forces many poultry farmers to rely on traditional manual incubation methods, which are not only labor-intensive but also highly inefficient.

To address this issue, our study focuses on designing and constructing an automatic egg incubator using locally available materials, optimized techniques, and straightforward modern technologies. By employing a prototyping approach, we successfully developed a cost-effective functional incubator. Our findings highlight its effectiveness in maintaining consistent temperature and humidity levels, which are essential for optimal egg hatching. This suggests that our incubator could serve as a reliable and effective tool for hatching poultry eggs.

When comparing our prototype incubator with commercially available models, it becomes evident that our prototype offers a cost-effective, accessible, and reliable solution tailored to the needs of local poultry farmers. Its affordability, reliance on locally available materials, and user-friendly design make it a practical choice for regions with limited resources. While commercial incubators may offer advanced features and higher precision, the prototype’s simplicity, adaptability, and cost efficiency position it as a valuable tool for enhancing poultry farming in Cameroon.

To further optimize its performance, we recommend that future research should focus on identifying the best conditions for maximizing hatching efficiency and consider the integration of remote-control features. This would enable the operator to remotely access the conditions in the incubator as well as to switch them on or off, enhancing convenience.

## Supporting information

S1 Text Arduino code of our incubator. (DOCX)
