# Supplementary material for: Customizing egg incubators for Cameroon: A design and construction guide
Source: PLoS One. 2025 May 15;20(5):e0322357. doi: 10.1371/journal.pone.0322357 (PMC12080822; doi:10.1371/journal.pone.0322357)
Supplement: S1 Text — (DOCX) [file pone.0322357.s001.docx]

# **Arduino code of our Incubator**

#include <Wire.h>

#include<LiquidCrystal_I2C.h>

#include<SimpleDHT.h> // DHT11

int pinDHT11 = 2 ;

SimpleDHT11 dht11 (pinDHT11 ) ;

LiquidCrystal_I2C lcd (0x3F, 20, 4 ) ;  // Set the LCD address to 0x3F for 20 chars and 4 lines

// Initialization of global variables

int humidity ;

int temperature ;

int humidity_user ;

int temperature_user ;

String turnerState ;

int turnerM = 0 ;

// Input & Button Logic

const int numOfInputs = 4 ;

const int numOfInputs1 = 3 ;

const int inputPins [ numOfInputs ] = { 8 , 9 , 10 , 11 }; // Menu buttons

const int inputPins1 [ numOfInputs1 ] = { 0 , 1 2 , 1 3 }; // Turner buttons

int inputState [ numOfInputs ] ;

int lastInputState [ numOfInputs ] = {LOW, LOW, LOW, LOW} ;

bool inputFlags [ numOfInputs ] = {LOW, LOW, LOW, LOW} ;

long lastDebounceTime [ numOfInputs ] = { 0 ,0 ,0 ,0 };

long debounceDelay = 1 ;

//LCD Menu Logic

const int numOfScreens = 4 ;

int currentScreen = 0 ;

String screens [ numOfScreens ][2] = {{" Temperature " ," ’C"} , {"Humidity " , "%"}, {"Turner " , " "},

{"Menu" , " " }};

int parameters [ numOfScreens ] = {30 ,70 ,0 ,0 };

// RELAYS

#define alarmRelay 1 // Digital Pin 1 to alarm Relay Module − alarm

#define heaterRelay 3 // Digital Pin 3 to h e at er Relay Module − h e at er

#define humidityRelay 4 // Digital Pin 4 to Humidity Relay Module − fan

#define motorRelayF 5 // Digital Pin 5 to motor Relay Module − motor

#define motorRelayB 6 // Digital Pin 6 to motor Relay Module − motor

#define exhaustRelay 7 // Digital Pin 7 to exhaust Relay Module − fan

void setup () {

    // declare all relays as outputs

    pinMode ( alarmRelay , OUTPUT) ;

    pinMode ( heaterRelay , OUTPUT) ;

    pinMode ( humidityRelay , OUTPUT) ;

    pinMode ( motorRelayF , OUTPUT) ;

    pinMode ( motorRelayB , OUTPUT) ;

    pinMode ( exhaustRelay , OUTPUT) ;

    // turn OFF any power to the relay channels during startup

    digitalWrite ( alarmRelay ,HIGH) ;

    digitalWrite ( heaterRelay ,HIGH) ;

    digitalWrite ( humidityRelay ,HIGH) ;

    digitalWrite ( motorRelayF ,HIGH) ;

    digitalWrite ( motorRelayB ,HIGH) ;

    digitalWrite ( exhaustRelay ,HIGH) ;

    for ( int i = 0 ; i < numOfInputs ; i++) {

        pinMode ( inputPins [i] , INPUT ) ;

        digitalWrite ( inputPins [i] , HIGH) ; // pull −up or use pinMode ( inputPins[i] , INPUT_PULLUP)

    }

    for ( int i = 0 ; i < numOfInputs1 ; i++) {

        pinMode ( inputPins1 [i] , INPUT ) ;

        digitalWrite ( inputPins1 [i] , HIGH) ; // pull −up

    }

    // Serial.begin ( 9600 ) ;

    lcd.begin () ;

    lcd.backlight() ;

    lcd.clear() ;

    lcd.setCursor ( 3 , 0 ) ;

    lcd.print(" EGG INCUBATOR" ) ;

    lcd.setCursor ( 0 , 1 ) ;

    lcd.print(" Powered by KEMEGNE M" ) ;

    lcd.setCursor( 3 , 2 ) ;

    lcd.print("COMPUTER SCIENCE" ) ;

    lcd.setCursor( 6 , 3 ) ;

    lcd.print(" IC 600 ( I I ) " ) ;

    delay( 5000 ) ;

    lcd.clear() ;

}

void loop () {

    static unsignedlong int sensorTime = 0;

    static bool startedDHT11 = false ;

    if ( millis () − sensorTime > 1500) {

        sensorTime = millis () ;

        // read without samples .

        byte temperature1 = 0 ;

        byte humidity1 = 0 ;

        int err = SimpleDHTErrSuccess ;

        if ( ( err = dht11 . read(&temperature1 , &humidity1 , NULL) ) != SimpleDHTErrSuccess ) {

            Serial.print("Read DHT11 failed , err ="); Serial. println ( err ) ; delay ( 1000 ) ;

            return ;

        }

        temperature = ( int ) temperature1 ;

        humidity = ( int ) humidity1 ;

        startedDHT11=true ;

    }else

        if ( startedDHT11 && millis () − sensorTime > 5) // 5mSec

        {

            // read sensor

            if ( currentScreen == 3){

                lcd.clear() ;

                mainScreenDisplay () ;

            }

            startedDHT11 = false ;

        }

    setInputFlags () ;

    resolveInputFlags () ;

    checkAndSwitchRelays () ;

}

void mainScreenDisplay () {

    displayReadings () ;

}

void checkAndSwitchRelays () { // compare sensor values with pot values to switch relays

    //Temperature control

    if ( temperature_user <= temperature )

    {

        // switch relay heater off

        digitalWrite( heaterRelay, HIGH) ;

    }

    else if ( temperature_user > temperature )

    {

        // switch relay heater on

        digitalWrite( heaterRelay, LOW) ;

    }

    //Humidity control

    if ( humidity_user <= humidity )

    {

        // switch relay fan off

        digitalWrite ( humidityRelay ,HIGH) ;

    }

    else if ( humidity_user > humidity )

    {

        // swit ch r e l a y fan on

        digitalWrite ( humidityRelay ,LOW) ;

    }

    // Exhauast fan control

    if ( ( humidity > ( humidity_user + 10)) | | ( temperature > ( temperature_user + 5 ) ) ){

        // switch relay fan on

        digitalWrite ( exhaustRelay ,LOW) ;

    } else {

        // switch relay fan off

        digitalWrite ( exhaustRelay ,HIGH) ;

    }

    if ( ( humidity > ( humidity_user + 15)) | | ( temperature > ( temperature_user + 10)) ){

        // switch relay alarm on

        digitalWrite( alarmRelay ,LOW) ;

    } else {

        // switch relay alarm off

        digitalWrite ( alarmRelay ,HIGH) ;

    }

    if (digitalRead ( inputPins1 [0] ) == LOW){

        turnerM = 1 ;

        if (digitalRead ( inputPins1[1] ) == LOW){

            digitalWrite ( motorRelayB ,LOW) ;

        } else if (digitalRead ( inputPins1[1] ) == HIGH){

            digitalWrite (motorRelayB ,HIGH) ;

        }

        if (digitalRead ( inputPins1[2] ) == LOW){

            digitalWrite(motorRelayF ,LOW) ;

        } else if( digitalRead ( inputPins1[2] ) == HIGH){

            digitalWrite(motorRelayF ,HIGH) ;

        }

    } else {

        turnerM = 0 ;

    }

}

void displayReadings () {

    turnerValue () ;

    lcd.clear () ;

    lcd.setCursor ( 0 , 0 ) ;

    lcd.print("Temp: " ) ;

    lcd.print( temperature ) ; lcd.print( " ’C" ) ;

    lcd.setCursor (10 , 0 ) ;

    lcd.print(" Set : " ) ;

    lcd.print( temperature_user ) ; lcd.print( " ’C" ) ;

    lcd.setCursor ( 0 , 1 ) ;

    lcd.print("RH: " ) ;

    lcd.print( humidity ) ; lcd.print("%");

    lcd.setCursor (11 , 1 ) ;

    lcd.print("Hum: " ) ;

    lcd.print( humidity_user ) ; lcd.print("%");

    lcd.setCursor ( 0 , 3 ) ;

    lcd.print(" Turner : " ) ;

    if ( turnerM == 1){

        lcd.print("Manual " ) ;

    } else {

        lcd.print( tu rn e rS t a t e ) ;

    }

    // Serial.print(" Humidity : " ) ;

    // Serial.print( humidity ) ;

    // Serial.println(" %\t " ) ;

    // Serial.print(" Temperature : " ) ;

    // Serial.print( temperature ) ;

    // Serial.println(" ∗C " ) ;

}

void setInputFlags () {

    for ( int i = 0 ; i < numOfInputs ; i++) {

        int reading = digitalRead ( inputPins [i] ) ;

        if ( reading != lastInputState [i] ) {

            lastDebounceTime [i] = millis() ;

        }

        if ( ( millis() − lastDebounceTime [i] ) > debounceDelay ) {

            if ( reading != inputState [i] ) {

                inputState [i] = reading;

                if ( inputState [i] == HIGH) {

                    inputFlags [i] = HIGH;

                }

            }

        }

        lastInputState [i] = reading;

    }

}

void resolveInputFlags () {

    for (int i = 0 ; i < numOfInputs ; i++) {

        if (inputFlags [i] == HIGH) {

            inputAction (i) ;

            inputFlags [i] = LOW;

            printScreen () ;

        }

    }

}

void inputAction ( int input ) {

    if ( input == 0) {

        if ( currentScreen == numOfScreens − 1){

        currentScreen = 0 ;

        } else {

        currentScreen++;

        }

    } else if ( input == 1){

        if ( currentScreen == 0){

        currentScreen = numOfScreens − 1;

        } else {

        currentScreen −−;

        }

    } else if ( input == 2) {

        parameterChange ( 0 ) ;

    } else if ( input == 3) {

        parameterChange ( 1 ) ;

    }

    temperature_user = ( parameters [ 0 ] ) ;

    humidity_user = ( parameters [ 1 ] ) ;

}

void parameterChange ( int key ) {

    if ( key == 0) {

        if ( currentScreen == 3){

        } else if ( currentScreen == 2){

        if ( parameters [ currentScreen ] < 2){

            parameters [ currentScreen ]++;

        }

        } else {

            parameters [ currentScreen ]++;

        }

    } else if ( key == 1) {

        if ( currentScreen == 3){

        } else {

            if ( parameters [ currentScreen ] == 0){

            } else {

                parameters [ currentScreen ]−−;

            }

        }

    }

}

void turnerValue () {

    if ( parameters[2] == 0){

        turnerState = "On" ;

    } else if ( parameters[2] == 1){

        turnerState = " off " ;

    }

}

void printScreen () {

    if ( currentScreen == 3){

    displayR eadings () ;

    } else

    if ( currentScreen == 2){

        turnerValue () ;

        lcd.clear () ;

        lcd.print ("∗∗∗∗∗∗∗ Menu ∗∗∗∗∗∗");

        lcd.setCursor ( 0 , 2 ) ;

        lcd.print ( screens [ currentScreen ] [ 0 ] ) ;

        lcd.print ( " : " ) ;

        lcd.setCursor ( 0 , 3 ) ;

        lcd.print ( turnerState ) ;

    } else {

        lcd.clear () ;

        lcd.print ("∗∗∗∗∗∗∗ Menu ∗∗∗∗∗∗∗");

        lcd.setCursor ( 0 , 2 ) ;

        lcd.print ( screens [ currentScreen ] [ 0 ] ) ;

        lcd.print ( " : " ) ;

        lcd.setCursor ( 0 , 3 ) ;

        lcd.print ( parameters [ currentScreen ] ) ;

        lcd.print (" " ) ;

        lcd.print ( screens [ currentScreen ] [ 1 ] ) ;

    }

}
